# *Sspdhx* Related to the Development and Virulence of *Sclerotinia sclerotiorum* Represents a Potential RNAi Target for Controlling Sclerotinia Disease

**DOI:** 10.1111/mpp.70244

**Published:** 2026-03-16

**Authors:** Qingna Shang, Shunrui Yang, Chunyu Feng, Chong Xie, Yunshu Song, Jiatao Xie, Yanping Fu, Jiasen Cheng, Qing Cai, Bo Li, Tao Chen, Xiao Yu, Yang Lin, Daohong Jiang, Xueqiong Xiao

**Affiliations:** ^1^ National Key Laboratory of Agricultural Microbiology Huazhong Agricultural University Wuhan China; ^2^ Hubei Key Laboratory of Plant Pathology, College of Plant Science and Technology Huazhong Agricultural University Wuhan China; ^3^ Hubei Hongshan Laboratory Wuhan China

**Keywords:** pyruvate dehydrogenase complex component X, RNA interference, *Sclerotinia sclerotiorum*, virulence

## Abstract

*Sclerotinia sclerotiorum* is a destructive pathogen with a broad host range, long‐term soil survival, and is difficult to control. Silencing virulence‐related genes is a strategy for controlling Sclerotinia disease. In this study, we identified and characterised *Sspdhx*, which encodes pyruvate dehydrogenase complex component X in *S. sclerotiorum*. *Sspdhx* deletion exhibited significant impairments in growth, sclerotia development, infection cushion formation, and virulence, indicating that *Sspdhx* plays important biological functions in *S. sclerotiorum*. *Sspdhx* deletion also resulted in reducing acetyl‐CoA and ATP levels, and increased sensitivity to multiple environmental stresses. Exogenous supplementation with acetyl‐L‐carnitine partially restored the virulence of the Δ*Sspdhx* mutants. Transcriptomic analyses revealed that deletion of *Sspdhx* disrupts central carbon metabolic homeostasis, leading to broad transcriptional reprogramming that affects genes involved in vegetative growth, stress adaptation, and virulence‐associated processes. Application of exogenous *Sspdhx*‐targeting dsRNA and host‐induced gene silencing in plants effectively silenced *Sspdhx* and attenuated the virulence of *S. sclerotiorum*. These findings potentially establish *Sspdhx* as a promising target for RNA‐based control strategies against Sclerotinia disease.

## Graphical abstract

SsPDHX affects acetyl‐CoA and ATP metabolism and is required for normal growth, development, and pathogenicity of *S. sclerotiorum*. SIGS or HIGS targeting *Sspdhx* enhances host resistance to Sclerotinia disease.

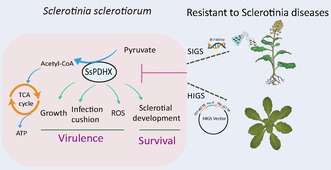

## 1 Introduction

Sclerotinia disease, caused by *Sclerotinia sclerotiorum*, is a devastating fungal disease with a wide host range, affecting many economically important crops such as *Brassica napus* , soybean, and sunflower, and leading to substantial agricultural losses (Boland and Hall 1994; Bolton et al. 2006; Yang, Liu, and Huang 2018). This phytopathogenic fungus mainly depends on sclerotia persisting in soil or plant debris to survive under unfavourable environmental conditions (Bolton et al. 2006; Brodal and Asdal 2021). Chemical control is widely employed for the management of Sclerotinia disease. However, resistance to fungicides has been observed in *S. sclerotiorum* under field conditions (Wang et al. 2014). Therefore, there is an increasing need to better understand the virulence mechanisms of *S. sclerotiorum* and identify novel virulence‐related genes, which are critical for developing resistant cultivars, eco‐friendly biocontrol strategies, and precision agriculture management.

*Sclerotinia sclerotiorum* employs a sophisticated pathogenic strategy. During the early stages of infection, its hyphal tips swell and branch extensively to form infection cushions that facilitate host penetration (Bary 1887; Lumsden and Dow 1973; Purdy 1958). This phytopathogenic fungus also secretes a diverse array of virulence factors, including cell wal‐degrading enzymes (CWDEs), cutinases, proteases, oxalic acid, and effectors, that act synergistically to disrupt host cell walls, suppress plant immunity, and promote colonisation (Liu et al. 2025; Shang et al. 2024). In response, plants have evolved diverse defence mechanisms mediated by receptor proteins that recognise pathogen‐derived signals (Ngou et al. 2022). This recognition induces a cascade of immune responses, including the rapid generation of reactive oxygen species (ROS), activation of mitogen‐activated protein kinase (MAPK) pathways, and transcriptional upregulation of defence‐related genes (Yu et al. 2017).

During plant–pathogen interactions, small RNAs (sRNAs) are encapsulated into extracellular vesicles and delivered into pathogenic fungi where they silence virulence‐related genes (Cai et al. 2018; Huang et al. 2019). This natural process has inspired two main biotechnological applications. In host‐induced gene silencing (HIGS), plants are engineered to produce sRNAs or double‐stranded (ds) RNAs that target fungal virulence‐related genes, thereby conferring resistance to infection (Song and Thomma 2018; Wang et al. 2017). Similarly, spray‐induced gene silencing (SIGS) exploits the ability of pathogens to take up environmental dsRNAs, which are then processed into sRNAs to silence target genes (Qiao et al. 2021). To date, these two technologies (HIGS and SIGS) have demonstrated efficacy against multiple crop diseases, including rice bakanae disease (Hou et al. 2025), sugarcane pokkah boeng disease (Yin et al. 2025), and wheat stripe rust (Qi et al. 2018). Although recent studies report successful application of HIGS and SIGS for managing Sclerotinia disease (Han et al. 2024; Zhang et al. 2025), the number of validated target genes for effective control remains limited.

In eukaryotes, the pyruvate dehydrogenase complex (PDC) is a large mitochondrial multienzyme complex centered on three catalytic components—pyruvate dehydrogenase (E1), dihydrolipoamide acetyltransferase (E2), and dihydrolipoamide dehydrogenase (E3)—together with additional non‐catalytic components required for proper assembly and regulation. This complex catalyses the oxidative decarboxylation of pyruvate, a process that requires multiple cofactors, including thiamine pyrophosphate, lipoic acid, FAD, NAD^+^, and coenzyme A (Izard et al. 1999; Patel and Roche 1990). By catalysing the conversion of pyruvate into acetyl‐CoA, PDC connects glycolysis with the tricarboxylic acid (TCA) cycle and thereby supports mitochondrial oxidative phosphorylation (Patel and Roche 1990). During oxidative phosphorylation, electron leakage from the electron transport chain (ETC) constitutes a major source of intracellular ROS (Kuznetsov et al. 2022), linking mitochondrial metabolic activity to cellular redox homeostasis. Consistent with this connection, perturbation of PDC activity has been shown to affect ROS balance and fungal pathogenicity. In *Fusarium graminearum*, genetic deletion of pyruvate dehydrogenase kinase 1 (FgPDK1), a key regulator of PDC activity, results in excessive ROS accumulation, plasma membrane disruption, and reduced virulence, highlighting the importance of PDC‐mediated metabolic regulation during host infection (Gao et al. 2016). Owing to its central role in primary metabolism, components of the PDC, including the pyruvate dehydrogenase E1 subunit, have been proposed as potential targets for the development of fungicides and herbicides (He et al. 2011; Zhou et al. 2021).

Beyond the catalytic core, proper assembly and function of the PDC also depend on specific non‐catalytic components. Incorporation of the E3 subunit into the PDC is mediated by the E3‐binding protein (E3BP), also termed pyruvate dehydrogenase complex component X (PDHX). Loss of PDHX disrupts PDC assembly and pyruvate metabolism, supporting its essential structural role in maintaining PDC integrity and metabolic flux (Gray et al. 2014; Jiang et al. 2025). Given its central role in carbon metabolism, PDHX is generally considered a basic metabolic component that likely contributes to maintaining cellular energy homeostasis. In mammals, PDHX dysfunction has been associated with a range of metabolic disorders and diseases (Brown et al. 2002; Huang et al. 2023; Jiang et al. 2025). However, fungal PDCs exhibit structural divergence from mammalian complexes (Forsberg 2023; Forsberg et al. 2020), and the biological functions of PDHX homologues in pathogenic fungi remain poorly understood. In this study, we investigated the biological function of the PDHX homologue in *S. sclerotiorum* and assessed its potential as a target for disease management by two strategies of SIGS and HIGS.

## 2 Results

### 2.1 Identification of Pyruvate Dehydrogenase Complex X in *S. sclerotiorum*

**FIGURE 1:**
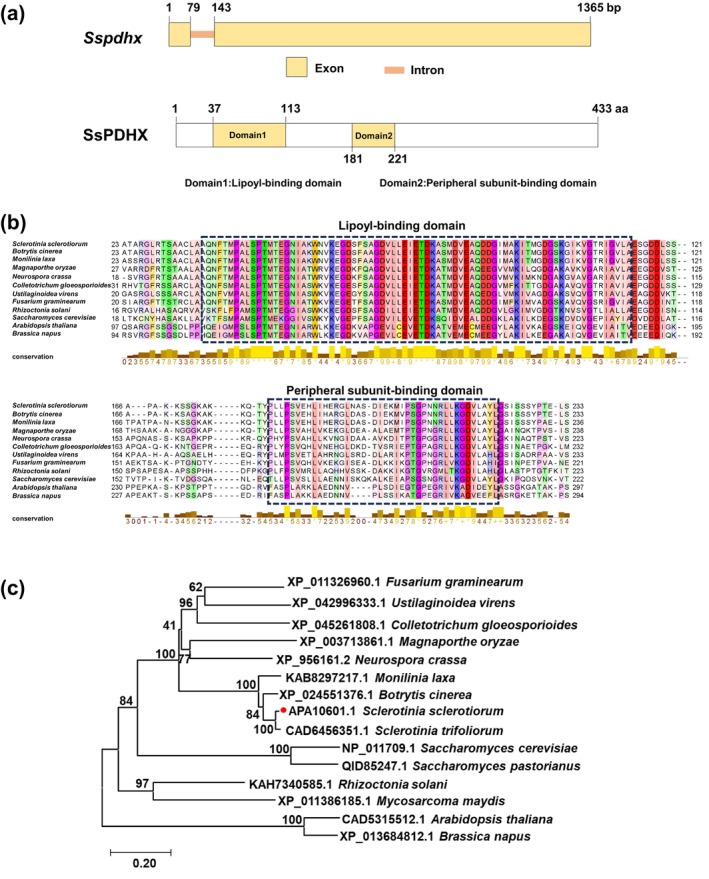
Structural, multiple alignment, and phylogenetic analysis of *Sspdhx* in *Sclerotinia sclerotiorum*. (a) The structures of *Sspdhx* gene (upper panel) and its encoding protein SsPDHX (lower panel). SsPDHX with 433 amino acids contains a lipoyl‐binding domain and a peripheral subunit‐binding domain (PSBD). (b) Multiple sequence alignment of SsPDHX with homologues from 11 fungi species. Conserved domains are highlighted with black dashed boxes. Conservation score is visualised as a histogram. Fully conserved columns are indicated by an asterisk (*) (score of 11 with the default amino acid property grouping), and columns containing substitutions in which all physicochemical properties are conserved are marked with a plus sign (+) (score of 10). (c) Phylogenetic tree of SsPDHX and its homologues were constructed using the neighbour‐joining method in MEGA 11. Branch lengths are proportional to the average probability of characteristic changes. The *S. sclerotiorum* sequence (SsPDHX) is highlighted with a red circle.

The *Sspdhx* sequence from the experimental Sunf‐M isolate matches the 1980 reference sequence (*sscle_06g053710*), which encodes pyruvate dehydrogenase complex component X (PDHX) in *S. sclerotiorum*. The gene is 1365 bp in length excluding the 5′‐and 3′‐untranslated regions (UTRs), and comprises two exons and one intron. It encodes a 433‐amino acid protein with a predicted molecular weight of 46.5 kDa, which is hypothesised to function as the E3‐binding protein of the PDC. SsPDHX possesses a predicted lipoyl‐binding domain and a peripheral subunit‐binding domain (PSBD) (Figure 1a). The lipoyl‐binding domain functions as a flexible arm to transfer acyl groups and electrons (Reed 2001), whereas the PSBD anchors E1 or E3 to the PDC (Patel et al. 2014), thereby ensuring coordinated catalysis and proper assembly of the complex. Sequence alignment analysis showed that the lipoyl‐binding domain and PSBD of SsPDHX are highly conserved across multiple species (Figure 1b). Phylogenetic analysis indicates that SsPDHX is evolutionarily conserved among Ascomycota fungi (Figure 1c).

Two homozygous knockout mutants of gene *Sspdhx* (Δ*Sspdhx‐19*, Δ*Sspdhx‐20*) and one complemented strain (*Sspdhx*‐19C) were generated, which were confirmed by PCR and reverse transcription (RT)‐PCR analyses. Southern blotting verified single‐copy integration of the hygromycin resistance gene cassette in the knockout mutants (Figure S1).

### 2.2 SsPDHX Is Required for Pathogenicity and Infection Cushion Formation

**FIGURE 2:**
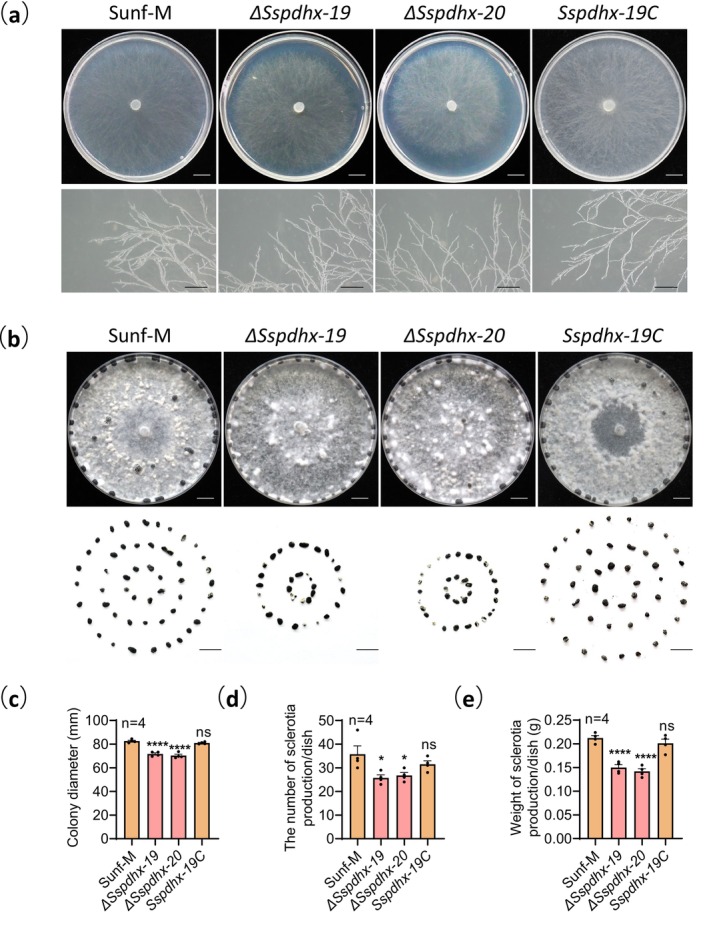
Deletion of *Sspdhx* significantly affects growth and sclerotial development in *Sclerotinia sclerotiorum*. (a) Colony morphology and hyphal tips of strain Sunf‐M and Δ*Sspdhx* mutants (potato dextrose agar [PDA], 20°C). Upper panel scale bar, 1 cm; lower panel scale bar, 1 mm. (b) Sclerotial morphology of strain Sunf‐M and Δ*Sspdhx* mutants (PDA, 14 days, 20°C). Sclerotia were collected from four 9‐cm PDA plates. Scale bar, 1 cm. (c) Colony diameters of strain Sunf‐M and Δ*Sspdhx* mutants on PDA at 20°C for 48 h. (d) Number and (e) dry weight of sclerotia produced by strain Sunf‐M and Δ*Sspdhx* mutants. Data were analysed by one‐way ANOVA; *n* indicates the number of independent replicates, and error bars represent standard error. Asterisks (*) denote significant differences (*p < 0.05, ****p < 0.0001), ns indicates no significant difference.

To investigate the role of *Sspdhx* in *S. sclerotiorum*, the phenotypefs of knockout mutants, complemented strain, and the wild‐type strain Sunf‐M were compared. Colony morphology and hyphal tip structure showed no obvious differences (Figure 2a). However, the growth rate of Δ*Sspdhx* mutants was reduced by approximately 14.0% (Figure 2c). After 14 days of culture, the Δ*Sspdhx* mutants produced approximately 26.6% fewer sclerotia and exhibited a 31.3% reduction in sclerotial dry weight compared with strain Sunf‐M (Figure 2b,d,e).

**FIGURE 3:**
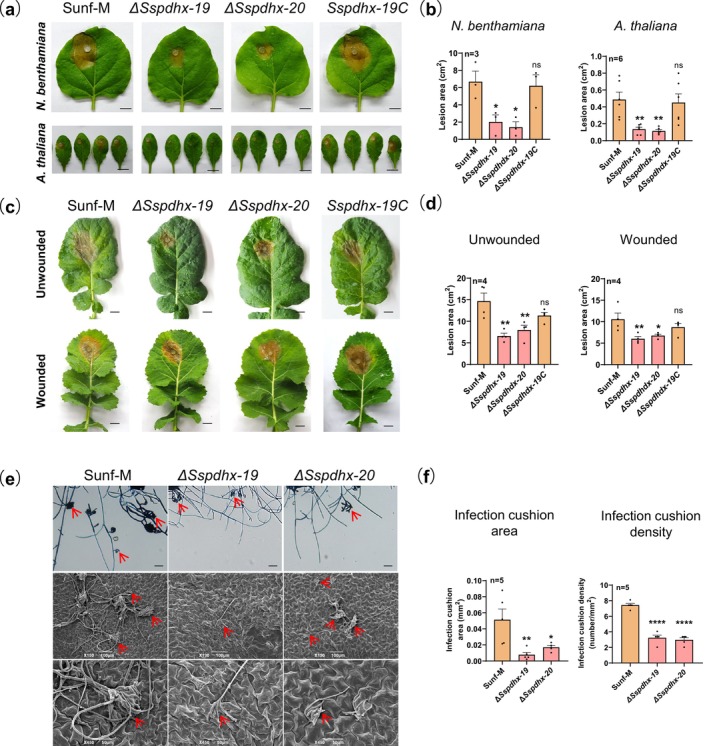
Deletion of *Sspdhx* impairs virulence and infection cushion formation of *Sclerotinia sclerotiorum*. (a) Virulence assays of strain Sunf‐M and Δ*Sspdhx* mutants on *Nicotiana benthamiana* and *Arabidopsis thaliana* leaves. Inoculated leaves were incubated at 20°C and photographed at 48 h post‐inoculation (hpi). Scale bar, 1 cm. (b) Quantification of lesions caused by the Sunf‐M and Δ*Sspdhx* mutants. (c) Virulence assays on unwounded or wounded *Brassica napus* leaves. Wounded *B. napus* were photographed at 48 hpi, and unwounded *B. napus* leaves were photographed at 72 hpi. Scale bar, 1 cm. (d) Lesion areas caused by Sunf‐M and Δ*Sspdhx* mutants on *B. napus* leaves. (e) Infection cushions of Sunf‐M and Δ*Sspdhx* mutants on glass slides (24 hpi) and *A. thaliana* leaves (8 hpi). Upper and middle panel scale bar, 100 μm; lower panel scale bar, 50 μm. (f) Infection cushion area and density on glass slides quantified using ImageJ. Data were analysed by one‐way ANOVA; *n* indicates the number of independent replicates, and error bars represent standard error. Asterisks (*) denote significant differences (*p < 0.05, **p < 0.01, ****p < 0.0001), ns indicates no significant difference.

To investigate the function of *Sspdhx* in the virulence of *S. sclerotiorum*, we examined the virulence of *Sspdhx* knockout mutants on *Nicotiana benthamiana*, *Arabidopsis thaliana* , and *B. napus* . Compared to strain Sunf‐M, Δ*Sspdhx* mutants exhibited significantly reduced virulence on all three host plants, while the complemented strain *Sspdhx‐19C* restored virulence to strain Sunf‐M levels (Figure 3a–d). On *N. benthamiana* leaves at 2 days post‐inoculation (dpi), the lesion areas caused by two Δ*Sspdhx* mutants (2.0 ± 0.8 cm^2^ and 1.4 ± 0.6 cm^2^) were smaller than that caused by the strain Sunf‐M (6.7 ± 1.2 cm^2^) and complemented strain *Sspdhx‐19C* (6.2 ± 1.3 cm^2^). Similarly, on *A. thaliana* leaves at 2 dpi, the Δ*Sspdhx* mutants produced lesions of 0.14 ± 0.02 cm^2^ and 0.12 ± 0.02 cm^2^, compared to 0.49 ± 0.08 cm^2^ and 0.45 ± 0.10 cm^2^ for Sunf‐M and *Sspdhx‐19C* (Figure 3a,b). We also evaluated the effect of wounding on *B. napus* leaves on the virulence of *Sspdhx* mutants. On intact *B. napus* leaves, the Δ*Sspdhx* mutants showed a 50.3% reduction in lesion area compared to strain Sunf‐M, whereas on wounded leaves, the reduction was 39.4% (Figure 3c,d).

To assess whether deletion of *Sspdhx* affects the penetration ability of *S. sclerotiorum*, we compared strain Sunf‐M and the Δ*Sspdhx* mutants by examining infection cushion formation on both glass slides and *A. thaliana* leaves. The results showed that the Δ*Sspdhx* mutants exhibited significantly reduced infection cushion numbers and sizes on both glass slides and *A. thaliana* leaves compared to strain Sunf‐M (Figure 3e). Specifically, the mutants formed 58.2% fewer infection cushions and showed an 82% reduction in infection cushion area on glass surfaces (Figure 3f).

### 2.3 *Sspdhx* Deletion Reduces Acetyl‐CoA and ATP Levels

**FIGURE 4:**
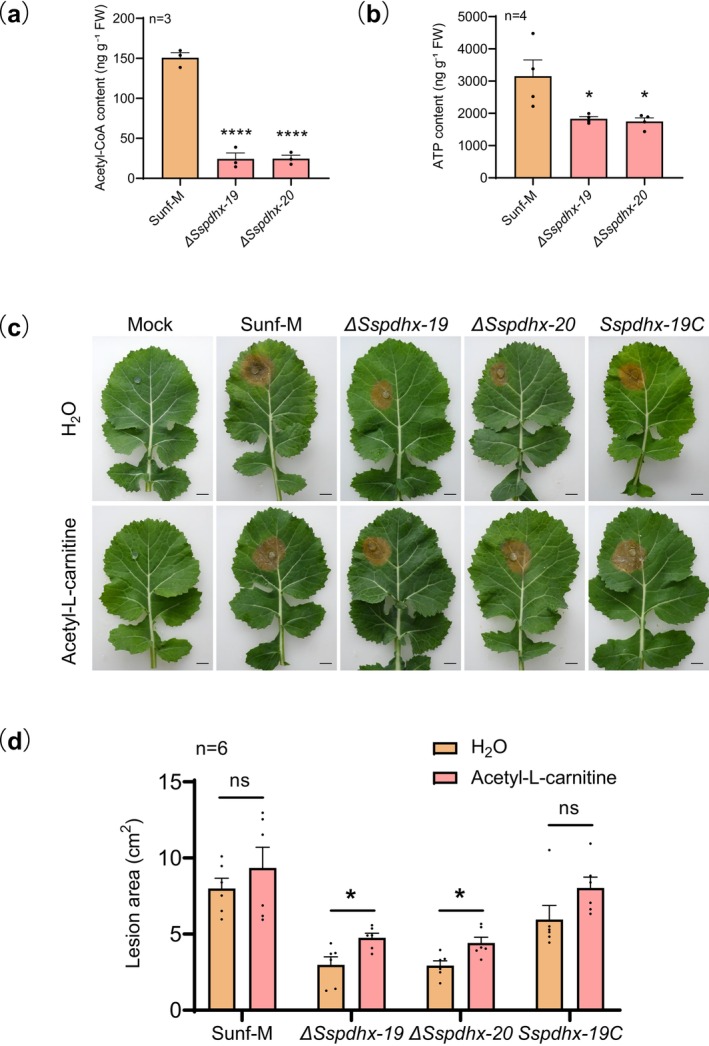
Deletion of *Sspdhx* reduced acetyl‐CoA and ATP levels in *Sclerotinia sclerotiorum*. (a) Acetyl‐CoA and (b) ATP levels in mycelia of strain Sunf‐M and *Sspdhx* deletion mutants. (c) Lesions on *Brassica napus* leaves at 3 days post‐inoculation with Sunf‐M, Δ*Sspdhx* mutants, and the complemented strain *Sspdhx‐19C* treated with water or 50 μM acetyl‐L‐carnitine. Mock indicates inoculation with agar plugs without mycelium. (d) Lesion areas quantified using ImageJ. Scale bar, 1 cm. Data were analysed by one‐way ANOVA; *n* indicates the number of independent replicates, and error bars represent standard error. Asterisks (*) denote significant differences (*p < 0.05, ****p < 0.0001).

As a key component of the PDC, SsPDHX could contribute to the conversion of pyruvate to acetyl‐CoA, a precursor for the TCA cycle. Therefore, we measured the effect of *Sspdhx* deletion on acetyl‐CoA and ATP levels in *S. sclerotiorum*. Compared to strain Sunf‐M, the *Sspdhx* deletions showed an 83.8% reduction in acetyl‐CoA and a 43.2% decrease in ATP level (Figure 4a,b).

To determine whether the reduced virulence of the Δ*Sspdhx* mutants was due to decreased acetyl‐CoA availability, exogenous acetyl‐L‐carnitine was applied to *B. napus* leaves prior to inoculation with *S. sclerotiorum*. After incubation at 20°C for 3 days, lesion areas were measured. Acetyl‐L‐carnitine supplementation significantly restored the virulence of mutants Δ*Sspdhx‐19* and Δ*Sspdhx‐20*, whereas no significant effect was observed in strain Sunf‐M and *Sspdhx‐19C* (Figure 4c,d). These findings suggest that acetyl‐CoA deficiency contributes to the loss of virulence in the *Sspdhx* deletion mutants, and that acetyl‐L‐carnitine can compensate for this metabolic limitation by replenishing the acetyl‐CoA pool.

### 2.4 Deletion of *Sspdhx* Compromises Stress Tolerance Under Multiple Conditions

The ability of *S. sclerotiorum* to regulate the host redox environment is critical for successful colonisation. During the early stages of infection, *S. sclerotiorum* suppresses the host oxidative burst to facilitate colonisation, whereas at later stages it induces ROS accumulation, thereby promoting host cell death and accelerating disease development (Kabbage et al. 2013; Williams et al. 2011). To evaluate the role of *Sspdhx* in oxidative stress tolerance, we examined the sensitivity of Δ*Sspdhx* mutants to exogenous H_2_O_2_. Compared with strain Sunf‐M, Δ*Sspdhx* mutants exhibited markedly increased sensitivity to H_2_O_2_. The average inhibition rate of 1 mM H_2_O_2_ was 40.6% for the Δ*Sspdhx* mutants, whereas it was only 22.2% for strain Sunf‐M. Furthermore, the Δ*Sspdhx* mutants showed almost no growth on potato dextrose agar (PDA) supplemented with 5 mM H_2_O_2_ (Figure S2). Moreover, we evaluated whether deletion of *Sspdhx* affects fungal tolerance to abiotic stresses. The results showed that gene *Sspdhx* deletion mutants were more sensitive than strain Sunf‐M to cell wall‐perturbing agents (Congo red) and membrane stress (SDS), as well as ionic stresses induced by CaCl_2_ and NaCl (Figure S3).

### 2.5 Deletion of *Sspdhx* Disrupts Central Metabolic Homeostasis

To investigate the molecular mechanism by which *Sspdhx* affects the phenotype of *S. sclerotiorum*, we performed transcriptomic profiling of the Δ*Sspdhx‐20* mutant and Sunf‐M strain during vegetative growth and at 16 hpi on *B. napus* . Principal component and correlation analyses confirmed high reproducibility (Figure S4a,b). A total of 1038 differentially expressed genes (DEGs) were identified during vegetative growth, and 778 DEGs were detected at 16 hpi (Figure S4c,d).

**FIGURE 5:**
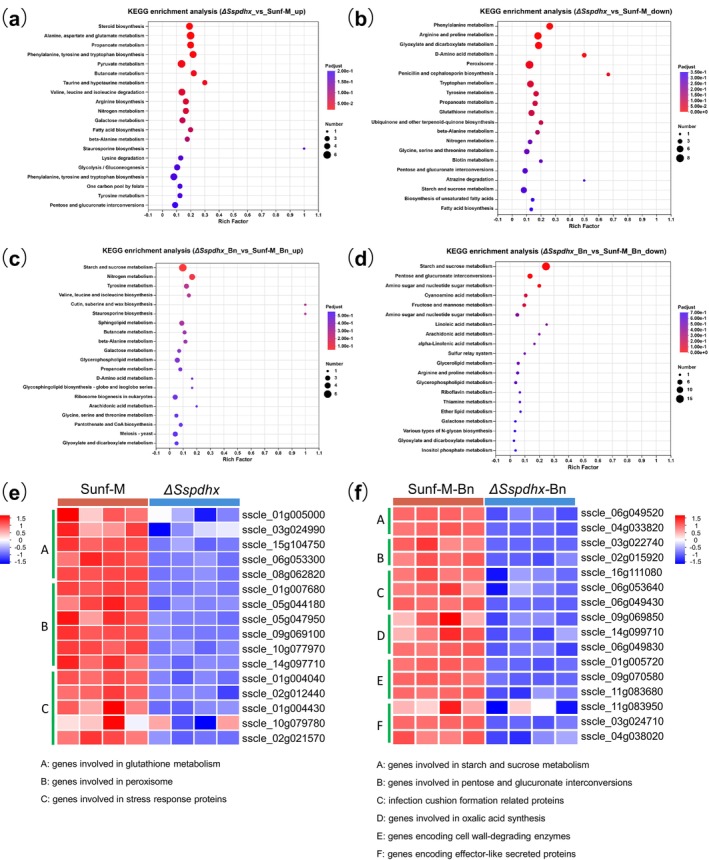
RNA‐seq analysis of differentially expressed genes (DEGs) between Sunf‐M and Δ*Sspdhx* during vegetative growth and infection stage. During vegetative growth, KEGG pathway enrichment analysis of genes upregulated (a) and downregulated (b) in Δ*Sspdhx* compared with Sunf‐M. During host infection, KEGG pathway enrichment analysis of genes upregulated (c) and downregulated (d) in Δ*Sspdhx* compared with Sunf‐M. (e) Heatmap showing the expression profiles of genes involved in glutathione metabolism, peroxisome function, and stress response in Sunf‐M and Δ*Sspdhx* during vegetative growth. (f) Heatmap showing the expression profiles of genes involved in starch and sucrose metabolism, pentose and glucuronate interconversions, and selected virulence‐related genes, including genes involved in infection cushion formation, oxalic acid biosynthesis, cell wall degradation, and effector‐like functions in Sunf‐M and Δ*Sspdhx‐20* during host infection.

During vegetative growth, GO enrichment analysis revealed that DEGs were mainly involved in carbohydrate metabolism, catalytic activity, and oxidoreductase activity, suggesting central metabolic disruptions due to *Sspdhx* deletion (Figure S4e). KEGG analysis showed alterations in amino acid metabolism, carbon metabolism, and redox‐related pathways, highlighting the role of *Sspdhx* in metabolic homeostasis, redox balance, and stress response (Figure 5a,b).

At 16 hpi, DEGs were enriched in categories related to polysaccharide binding, cellulose degradation, and membrane functions (Figure S4f), with significant changes in carbohydrate metabolism, including starch and sucrose pathways (Figure 5c,d). This reprogramming may impair the pathogen's ability to degrade host cell walls and adapt to the host, contributing to reduced virulence. Gene expression dynamics (Figure 5e,f), validated by RT‐quantitative PCR (qPCR) (Figure S5), revealed coordinated downregulation of genes involved in glutathione metabolism and stress responses during vegetative growth, as well as reduced expression of metabolism‐ and virulence‐related genes during infection.

### 2.6 Exogenous *Sspdhx*‐dsRNA Application Reduces Virulence of *S. sclerotiorum*

**FIGURE 6:**
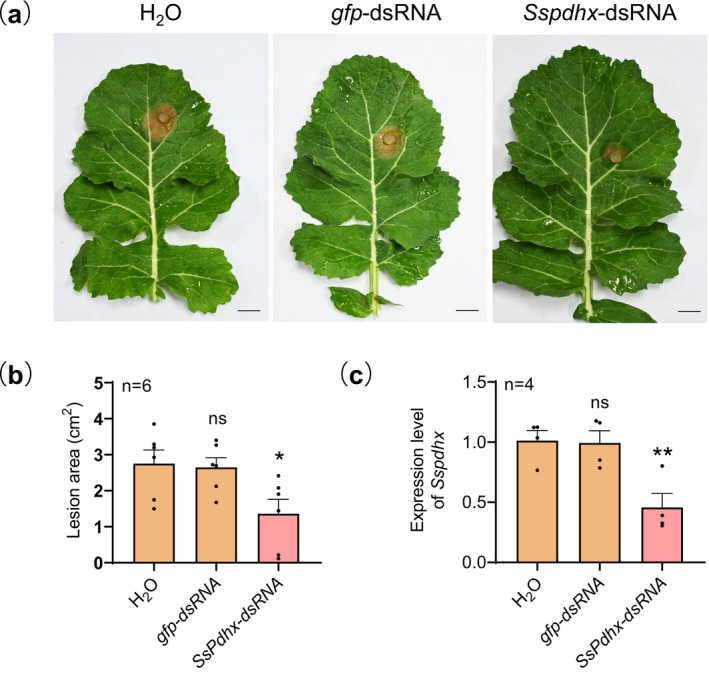
Exogenous application of *Sspdhx* dsRNA significantly reduces the virulence of *Sclerotinia sclerotiorum*. (a) Symptoms on *Brassica napus* leaves inoculated with *S. sclerotiorum* strain Sunf‐M after treatment with the in vitro‐synthesised double‐stranded (ds) RNA targeting *Sspdhx* (*Sspdhx*‐dsRNA), compared to treatments with water or *gfp*‐dsRNA. Photographs were taken at 48 h post‐inoculation (20°C). Scale bar, 1 cm. (b) Lesion areas measured using ImageJ. (c) Relative expression levels of *Sspdhx* in *S. sclerotiorum*. Mycelia were collected from *B. napus* leaves treated with double‐distilled water, *gfp*‐dsRNA, or *Sspdhx*‐dsRNA. The expression level of *Sspdhx* in mycelia from double‐distilled water‐treated plant was normalised to 1. The *β‐tubulin* gene in *S. sclerotiorum* was used as an internal control. Data were analysed by one‐way ANOVA; *n* indicates the number of independent replicates, and error bars represent standard error. Asterisks (*) denote significant differences (*p < 0.05, **p < 0.01), ns indicates no significant difference.

Because *Sspdhx* plays a crucial role in *S. sclerotiorum* growth and virulence, we applied exogenous dsRNA targeting *Sspdhx* to investigate its impact on the fungal virulence. Strain Sunf‐M was inoculated onto *B. napus* leaves pretreated with water, *gfp*‐dsRNA, or *Sspdhx*‐dsRNA, and lesion areas were measured. *Sspdhx*‐dsRNA treatment resulted in significantly smaller lesions compared to two controls (water or *gfp*‐dsRNA) (Figure 6a,b), which is consistent with markedly downregulated *Sspdhx* expression in *S. sclerotiorum* (Figure 6c). These findings indicate that *Sspdhx* could serve as an effective target for SIGS to control Sclerotinia disease.

### 2.7 HIGS of *Sspdhx* Enhances Resistance in *N. benthamiana* and *A. thaliana*

**FIGURE 7:**
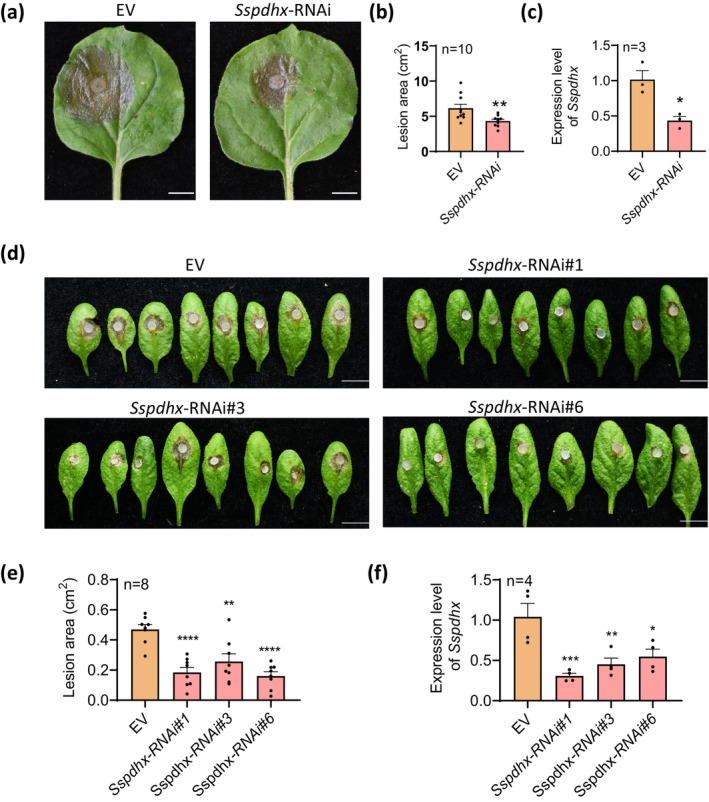
Host‐induced gene silencing (HIGS) of *Sspdhx* in *Nicotiana benthamiana* and *Arabidopsis thaliana* . (a) Virulence assay of strain Sunf‐M on *N. benthamiana* leaves transiently expressing the *Sspdhx*‐RNAi vector or empty vector (EV). Photographs were taken at 60 h post‐inoculation (20°C). Scale bar, 1 cm. (b) Lesions caused by strain Sunf‐M on *N. benthamiana* leaves transiently expressing the *Sspdhx*‐RNAi vector or EV. (c) *Sspdhx* expression level during *S. sclerotiorum* infection process on *N. benthamiana* leaves treated with *Sspdhx*‐RNAi or EV. (d) Virulence assay of strain Sunf‐M on transgenic *A. thaliana* plants expressing *Sspdhx*‐RNAi vector or EV. Scale bar, 1 cm. Photographs were taken after 36 h of incubation at 20°C. (e) Lesion caused by Sunf‐M on *A. thaliana* leaves. (f) Relative expression levels of *Sspdhx* in Sunf‐M during infection of *A. thaliana* leaves. The experiment was repeated twice with consistent results. Data were analysed by one‐way ANOVA; *n* represents the number of independent replicates, and error bars indicate standard error. Asterisks denote significant differences between groups (*p < 0.05, **p < 0.01, ***p < 0.001, ****p < 0.0001).

We selected a 307 bp fragment from the second exon of *Sspdhx* to construct an *Sspdhx*‐RNAi vector and expressed it in *N. benthamiana* and *A. thaliana* . This fragment is highly specific to the target gene *Sspdhx* with negligible off‐target effects in the host or non‐pathogens (Table S2). Transient expression of the *Sspdhx*‐RNAi construct in *N. benthamiana* led to significantly reduced lesion area (4.3 ± 0.2 cm^2^) compared to the empty vector (EV) control (6.1 ± 0.6 cm^2^) after inoculation with *S. sclerotiorum* (Figure 7a,b). RT‐qPCR confirmed *Sspdhx* expression was significantly suppressed in leaves expressing the *Sspdhx*‐RNAi vector (Figure 7c).

To evaluate the efficacy of HIGS in a stable genetic system, the *Sspdhx*‐RNAi construct was introduced into *A. thaliana* ecotype Col‐0. Sunf‐M caused significantly smaller lesions on transgenic *A. thaliana* lines compared with EV controls, confirming that HIGS‐mediated silencing of *Sspdhx* effectively compromises fungal virulence (Figure 7d–f).

## 3 Discussion

In this research, we identified an E3‐binding protein that serves as pyruvate dehydrogenase complex component X in *S. sclerotiorum*. The putative ortholog gene of *Sspdhx*, *PDX1*, functions as a structural scaffold of PDC in *Saccharomyces cerevisiae* , and deletion of *PDX1* abolishes PDC activity by preventing incorporation of the E3 component (Lawson et al. 1991), which is consistent with our finding that deletion of *Sspdhx* in *S. sclerotiorum* reduced acetyl‐CoA and ATP levels. In *Candida albicans* , disruption of *PDX1* causes a pronounced defect in filamentation, with cells largely confined to the yeast form, indicating the critical role of PDC integrity in fungal morphogenesis (Vellucci et al. 2007). Similarly, in *S. sclerotiorum*, although colony morphology was unaffected, *Sspdhx* deletion impaired infection cushion formation and reduced sclerotial production, both of which rely on proper hyphal differentiation (Li et al. 2018; Ordonez‐Valencia et al. 2015). Structural analyses have revealed that fungal E3BP (PDHX/PDX) differs from its mammalian counterpart; its biological functions in fungi, especially in plant‐pathogenic fungi, remain largely unexplored and require further investigation (Forsberg 2023; Forsberg et al. 2020). This study confirms that SsPDHX, a key component of the PDC, is involved in the growth, development, and pathogenicity of *S. sclerotiorum*. Infection cushions are known to be essential for *S. sclerotiorum* to penetrate the intact cuticle of host plants. When infecting through wounds, *S. sclerotiorum* directly penetrates the host via the wound site, bypassing the need for infection cushions (Xiao et al. 2014). Similarly, in this study, the lesion size caused by the Δ*Sspdhx* mutants on wounded hosts was larger than that on intact hosts, suggesting that defective infection cushion formation contributes to the virulence defect in the Δ*Sspdhx* mutants. Nevertheless, the mutant's virulence on wounded hosts did not fully restore to wild‐type levels, indicating that SsPDHX also plays a role in subsequent hyphal spread within the host after initial penetration.

PDC‐mediated acetyl‐CoA production is central to fungal metabolism and has been implicated in pathogenicity (Griffiths et al. 2012). The partial restoration of virulence upon exogenous acetyl‐L‐carnitine treatment supports that reduced acetyl‐CoA biosynthesis contributes to the Δ*Sspdhx* phenotype, potentially through disruption of energy metabolism and metabolite availability during host colonisation. The requirement for Ss‐Pth2, a carnitine acetyltransferase involved in acetyl‐CoA transport in *S. sclerotiorum*, further highlights the link between central metabolism and virulence (Liberti et al. 2013). Beyond defective in growth and acetyl‐CoA metabolism, Δ*Sspdhx* mutants exhibited increased sensitivity to multiple environmental stresses, particularly oxidative stress. This is consistent with a model in which SsPDHX maintains PDC stability and cellular redox balance, as PDC activity is known to be vulnerable to ROS‐mediated inactivation (Cabiscol et al. 2000; Samikkannu et al. 2003; Tabatabaie et al. 1996). The deletion of *Sspdhx* may therefore amplify oxidative stress, further compromising fungal fitness during infection. Transcriptomic analyses revealed broad reprogramming of carbohydrate metabolism and the downregulation of multiple virulence‐related genes, suggesting that the metabolic disruption caused by *Sspdhx* deletion has pleiotropic effects on growth, stress tolerance, and infection processes. Together, these results suggest that disruption of SsPDHX may affect PDC‐associated metabolic processes, thereby indirectly influencing multiple physiological and pathogenic traits in *S. sclerotiorum*.

**FIGURE 8:**
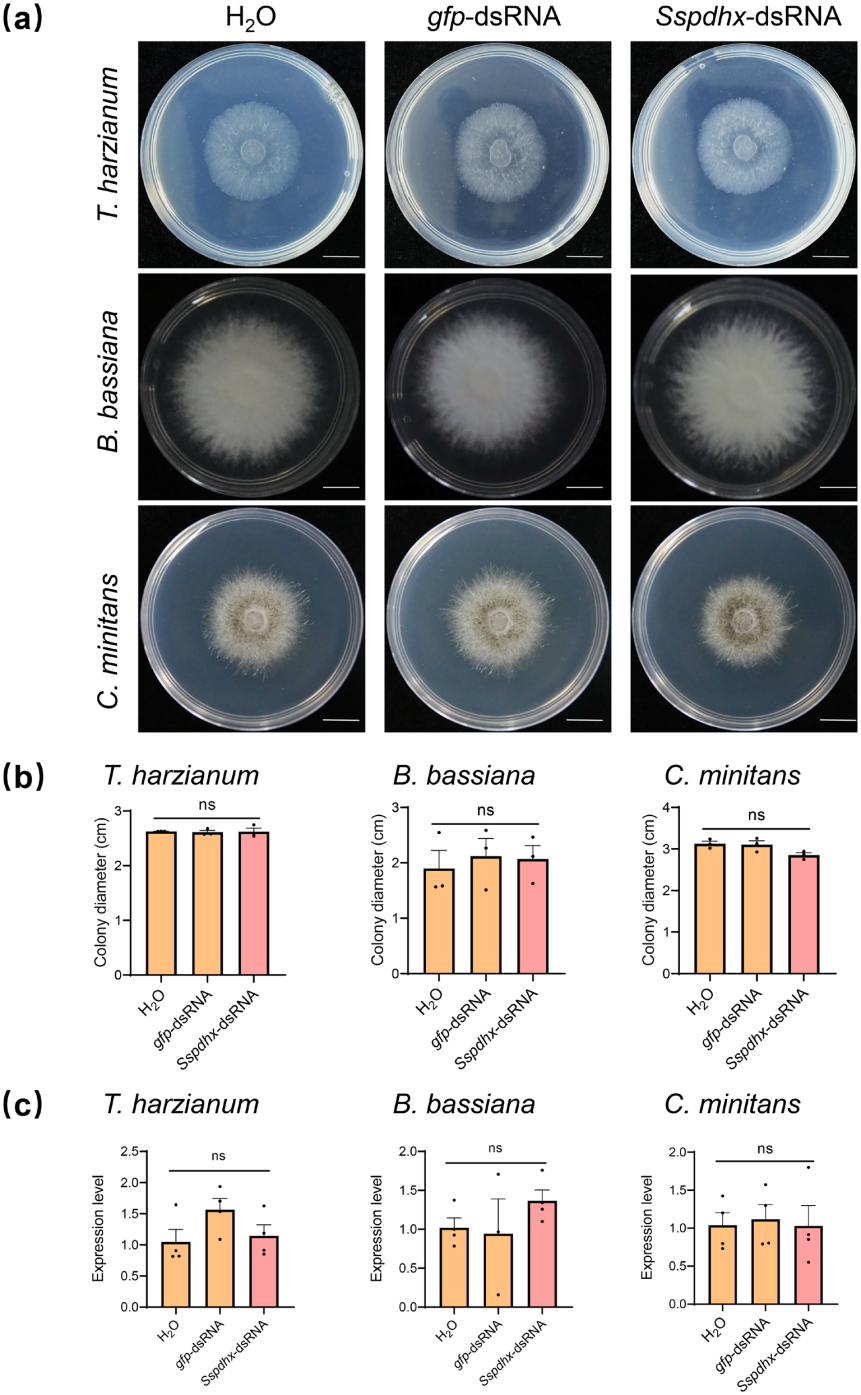
(a) Colony morphology and (b) Colony diameter of *Trichoderma harzianum* (20°C, 2 dpi), *Beauveria bassiana* (25°C, 7 dpi), and *Coniothyrium minitans* (20°C, 7 dpi) grown on potato dextrose agar (PDA) plates pretreated with *Sspdhx*‐dsRNA. Plates treated with water or *gfp*‐dsRNA served as controls. Scale bar, 1 cm. (c) Reverse transcription‐quantitative PCR analysis of *Sspdhx* homologues expression in *T. harzianum*, *B. bassiana*, and *C. minitans* cultured on PDA plates treated with *Sspdhx*‐dsRNA. *β‐tubulin*, *18S rRNA*, and *Actin* were used as reference genes for *T. harzianum*, *B. bassiana*, and *C. minitans*, respectively. Data were analysed by one‐way ANOVA, and error bars represent the standard error. ns indicates no significant difference compared with the water control (*p* > 0.05).

The carbendazim and dimethachlon‐resistant isolates of *S. sclerotiorum* have been reported (Wang et al. 2014). Thus, developing sustainable and environmentally friendly strategies is urgently needed for the effective management of Sclerotinia disease. RNAi‐based nucleic acid fungicides and transgenic plants may provide new opportunities to mitigate fungicide resistance (Koch and Kogel 2014; Wang and Jin 2017). Although field application remains challenged by RNA instability, delivery efficiency, and production costs, recent developments of carrier systems, chemical modification, sequence optimisation, and cost‐effective production platforms are helping to address these challenges (Cedden et al. 2025; Dalakouras et al. 2016; Wang et al. 2023). However, the identification of suitable and effective fungal target genes still represents a major limitation. For example, in *S. sclerotiorum*, only a limited number of candidate genes have been reported (Ouyang et al. 2025). To enable the practical development of RNAi‐based pesticides, it will be essential to target conserved and functionally indispensable genes, while avoiding targets that are prone to mutation or functional redundancy (Koch et al. 2019; Liu et al. 2011). Our findings identify SsPDHX as a promising target for novel control strategies against Sclerotinia disease. Because *S. sclerotiorum* can efficiently uptake exogenous dsRNA through clathrin‐mediated endocytosis (McLoughlin et al. 2018; Wytinck et al. 2020), RNAi targeting *Sspdhx* represents a feasible approach to suppress fungal pathogenicity. Importantly, deletion of *Sspdhx* reduces virulence and disrupts sclerotia development, indicating that silencing this gene may both attenuate infection and block the disease cycle. Unlike previously reported HIGS and SIGS targets in *S. sclerotiorum* (Ouyang et al. 2025), *Sspdhx* is essential for both virulence and survival, making it a particularly attractive RNAi target. It may serve as a potential strategy for controlling grey mould, while posing no harm to beneficial fungi such as *Trichoderma harzianum*, *Beauveria bassiana* and *Coniothyrium minitans* (Figure 8 and Table S2).

In summary, this study provides evidence that SsPDHX plays an important role in maintaining central metabolic functions, including acetyl‐CoA production and ATP generation, in *S. sclerotiorum*. Deletion of *Sspdhx* results in abnormal growth, sclerotial development, stress tolerance, and virulence, probably as a consequence of disrupted metabolic and redox homeostasis. In addition, the effective silencing of *Sspdhx* through both SIGS and HIGS highlights *Sspdhx* as a promising target for RNA‐based control strategies against Sclerotinia disease.

## 4 Experimental Procedures

### 4.1 Plants, Fungi, and Culture Conditions

*Sclerotinia sclerotiorum* strain Sunf‐M, isolated from a diseased sunflower, shows normal colony morphology on PDA (200 g/L potato, 20 g/L D‐glucose, 15 g/L agar) and strong virulence on host plants, including *B. napus* . All mutants were generated from strain Sunf‐M. Gene knockout mutants were selected and purified on PDA supplemented with hygromycin. Complementation strains were selected on PDA containing hygromycin and G418. All *S. sclerotiorum* strains were cultured on PDA at 20°C and stored on PDA slants at 4°C. *T. harzianum* and *C. minitans* were cultured on PDA at 20°C, while *B. bassiana* was cultured on PDA at 25°C.

*Arabidopsis thaliana* (Col‐0), *B. napus* , and *N. benthamiana* were grown in soil under controlled conditions. *A. thaliana* and *B. napus* were maintained at 22°C with a 12 h light/12 h dark cycle, whereas *N. benthamiana* was grown at 22°C–25°C with a 16 h light/8 h dark cycle.

### 4.2 Bioinformatics Analysis and Phylogenetic Analysis

The protein sequence of SsPDHX (sscle_06g053710) and its homologous from other species were retrieved from the NCBI GenBank database (https://www.ncbi.nlm.nih.gov/). The conserved functional domains in SsPDHX were predicted using UniProt (https://www.uniprot.org/). Multiple sequence alignment was performed using Jalview and the phylogenetic tree was constructed using the neighbour‐joining method in MEGA 11 with 1000 bootstrap replicates.

### 4.3 Gene Knockout and Complementation of *S. sclerotiorum*

The *Sspdhx* knockout mutants were generated using the split‐marker technique (Figure S1a) (Catlett et al. 2003). Briefly, approximately 1.5 kb upstream and downstream flanking sequences of *Sspdhx* were amplified from genomic DNA using primers *Sspdhx*‐UF/*Sspdhx*‐UR and *Sspdhx*‐DF/*Sspdhx*‐DR (Table S1). The hygromycin‐resistance gene was split into two fragments (HY and YG) with a 704 bp overlap, amplified from plasmid pUCH18. These fragments were fused by overlapping PCR. Using overlapping PCR, the upstream flanking sequence was fused to the HY fragment with primers *Sspdhx*‐UF/HY‐R, and the downstream flanking sequence was fused to the YG fragment with primers YG‐F/*Sspdhx*‐DR (Table S1). These two fusion fragments were co‐transformed into the protoplasts of *S. sclerotiorum* wild‐type strain Sunf‐M, with reference to the method described by Rollins (2003). The candidate knockout mutants were selected on regeneration medium containing 100 μg/mL hygromycin and initially screened by PCR.

For genetic complementation, the full‐length cDNA of *Sspdhx* was amplified and cloned into the pCETNS vector. The constructed plasmid was then introduced into the Δ*Sspdhx* mutants via *Agrobacterium*‐mediated transformation. Complemented strains were selected on PDA supplemented with 100 μg/mL hygromycin and 100 μg/mL G418, and successful complementation was confirmed through RT‐PCR.

### 4.4 Southern Blot Analysis

To confirm the copy number of hygromycin resistance cassette in putative knockout transformants, Southern blot analysis was performed. Mycelia were cultured in potato dextrose broth (PDB) for 2 days, harvested by centrifugation, washed twice with 0.7 M NaCl, and digested in an enzyme solution (10 mg/mL lysing enzymes from *T. harzianum* and 1 mg/mL snailase in 0.7 M NaCl) at 20°C for 2–3 h to release protoplasts. Protoplasts were filtered, collected by centrifugation, and subjected to genomic DNA extraction using a CTAB method. Briefly, protoplasts were lysed in 2% CTAB extraction buffer at 65°C for 25 min, and DNA was purified by phenol:chloroform:isoamyl alcohol (25:24:1) extraction, followed by chloroform:isoamyl alcohol (24:1) extraction. DNA was precipitated with ethanol, washed with 70% ethanol, and dissolved in RNase‐containing double‐distilled water. The genomic DNA was digested with XhoI, and analysed by Southern blot. An 800‐bp hygromycin resistance gene fragment was amplified using primers Hyg‐probe‐F/Hyg‐probe‐R (Table S1) as probe to confirm gene replacement. Probe labelling and hybridisation were performed with the AlkPhos Direct Labeling and Detection system (Cytiva).

### 4.5 Nucleic Acid Extraction and RT‐qPCR

Total RNA was extracted from mycelia grown on PDA or collected from plant tissues using TRIzol reagent. Following extraction, RNA was reverse transcribed into cDNA following the manufacturer's instructions of the cDNA synthesis supermix kit (TransGen Biotech). qPCR was performed with qPCR SuperMix (TransGen Biotech) on a QuantStudio 5 Real‐Time PCR system (Thermo Fisher Scientific). The relative expression was calculated by 2^−ΔΔ*C*t^ method, with *β‐tubulin* (*sscle_02g015170*) serving as the internal reference gene. RT‐qPCR analysis was performed with at least three biological replicates, each consisting of three technical replicates.

### 4.6 Phenotypic Characteristics

To assay fungal growth, mycelial plugs (5 mm in diameter) from strain Sunf‐M and knockout mutants were inoculated on the centre of PDA plates. Hyphal morphology was examined microscopically at 24 hpi, while colony expansion was quantified by measuring diameters at 48 hpi. Sclerotial development was assessed at 14 dpi, with sclerotia morphology, sclerotia quantity, and weight recorded. To assess fungal sensitivity to H_2_O_2_ and other abiotic stresses, mycelial plugs (5 mm in diameter) were inoculated onto PDA supplemented with H_2_O_2_ (1, 3, or 5 mM) or with Congo red (3 mg/mL), SDS (0.01%), CaCl_2_ (0.5 M), or NaCl (1 M). Colony diameters were measured after 2 days to calculate inhibition rates. All experiments were performed at least twice with three replicates each time.

The virulence of each strain was assessed by inoculating detached leaves of *A. thaliana* , *N. benthamiana*, or *B. napus* with mycelial plugs (2 mm or 5 mm in diameter). The inoculated leaves were incubated at 20°C for 48 or 72 h, after which lesion areas were measured. To assess the effect of acetyl‐L‐carnitine on the virulence of wild‐type *S. sclerotiorum* and Δ*Sspdhx* mutants, mycelial plugs were precultured on PDA containing 50 μM acetyl‐L‐carnitine (Sigma) for 36 h. Subsequently, 30 μL of 50 μM acetyl‐L‐carnitine solution was applied onto *B. napus* leaves, with water as the control. Leaves were then inoculated with Sunf‐M, Δ*Sspdhx‐19*, Δ*Sspdhx‐20*, or the complemented strain *Sspdhx‐19C*. Inoculation with agar plugs without mycelium was used as a negative control. Inoculated leaves were incubated at 20°C under humid conditions for 3 days, after which lesion areas were measured.

### 4.7 Infection Cushion Formation Assay

To investigate infection cushion formation, fresh mycelial plugs (5 mm in diameter) were inoculated onto glass slides and incubated at 20°C for 24 h. Samples were then stained with trypan blue staining solution (20% lactic acid, 20% phenol, 40% glycerol, 20% water, 0.05% trypan blue) for 2 h. After washing away the staining solution from the samples with water, the formation of infection cushions was observed under a microscope. The number and size of infection cushions were observed and quantified using ImageJ software (Xie et al. 2021).

For *in planta* observation, mycelial plugs (5 mm in diameter) were inoculated onto leaves of *A. thaliana* Col‐0 and incubated at 20°C for 8 h. Subsequently, the inoculated leaf tissues were dissected into 3 × 3 mm pieces (> 10 pieces per sample), immediately fixed in 2.5% glutaraldehyde and processed for scanning electron microscopy (SEM, SU3800, Hitachi) at the electron microscopy facility of Huazhong Agricultural University to examine infection cushions.

### 4.8 Detection of Acetyl‐CoA Formation and ATP Level

ATP levels in strain Sunf‐M and knockout mutants were performed using established protocols (Zhang et al. 2014). Fresh mycelia (20 mg) were collected, homogenised in lysis buffer, and centrifuged at 12,000 *g* for 10 min at 4°C. ATP concentrations in the supernatants were determined using an ATP Assay Kit (Beyotime Biotechnology) according to the manufacturer's instructions, and luminescence was recorded with a microplate reader.

Acetyl‐CoA was detected using the ELISA Kit (Sangon Biotech). Mycelia (0.1 g) were homogenised in phosphate‐buffered saline and centrifuged. The supernatant was incubated in coated wells with detection antibodies, washed, and developed for A_450_ measurement. All experiments were performed at least twice with three replicates each time.

### 4.9 Transcriptome Analysis

Fresh mycelia of *S. sclerotiorum* were cultured in PDB at 20°C with shaking for 2 days. For infection‐stage samples, 2‐day‐old mycelia were inoculated on *B. napus* leaves and then collected at 16 hpi. Total RNA was extracted with four independent biological replicates per treatment. RNA sequencing was performed on an Illumina NovaSeq X Plus platform (Majorbio). Clean reads were aligned to the *S. sclerotiorum* reference genome, and DEGs were identified using a fold change ≥ 2 and *p* ≤ 0.05. GO enrichment analysis was conducted using GOATOOLS with Fisher's exact test, and terms with adjusted *p* < 0.05 were considered significant. KEGG pathway enrichment analysis was performed in R, with adjusted *p* value < 0.05 as the significance threshold.

### 4.10 In Vitro Synthesis and Application of dsRNA

A 307‐bp fragment from the second exon of *Sspdhx* was selected as the target for SIGS. This fragment was amplified from *S. sclerotiorum* genomic DNA using KOD DNA polymerase (Toyobo) with primers containing T7 promoter sequences at the 5′ and 3′ ends (Table S1). The resulting PCR product was used for in vitro dsRNA synthesis with the In Vitro Transcription T7 Kit (Takara Bio Inc.). The primers used for in vitro dsRNA synthesis are listed in Table S1.

To evaluate the effect of *Sspdhx*‐dsRNA on the virulence of *S. sclerotiorum*, *B. napus* leaves were pretreated with 50 ng/μL dsRNA (containing 1‰ Tween 20) prior to inoculation with mycelial plugs (5 mm in diameter). Leaves treated with either double‐distilled water or *gfp*‐dsRNA served as controls. Lesion areas were measured at 48 hpi under 20°C.

### 4.11 Construction of HIGS Vector and Plant Transformation

For HIGS, the 307‐bp fragment from the second exon of *Sspdhx* was selected as the sense strand. The sense strand, together with the third intron (ms‐i3) from *A. thaliana* *malate synthase* (GenBank accession: AB005235) (Tinoco et al. 2010) and the corresponding antisense strand, was ligated into the plant expression vector pCNF3 (GenBank accession: MG733986) (Yang, Tang, et al. 2018) using a homologous recombination enzyme (Vazyme). The resulting construct was subsequently introduced into *Agrobacterium tumefaciens* GV3101.

To induce gene silencing in *N. benthamiana*, *A. tumefaciens* GV3101 carrying either the empty vector (EV) pCNF3 or the *Sspdhx*‐RNAi construct was infiltrated into 5‐week‐old *N. benthamiana* leaves at an OD_600_ of 0.8. Following infiltration, the plants were maintained in darkness for 3 days to facilitate RNAi construct expression. Subsequently, the leaves were inoculated with mycelial plugs (5 mm in diameter) of Sunf‐M and incubated at 20°C for 60 h, after which lesion areas were measured.

To obtain stable *A. thaliana* transformants, *A. tumefaciens* GV3101 carrying either the empty vector (EV) pCNF3 or the *Sspdhx*‐RNAi construct was used for floral dip transformation (Zhang et al. 2006). Transformed plants were selected on 1/2× Murashige and Skoog (MS) medium supplemented with 50 ng/μL kanamycin and further verified by PCR amplification. To assess the resistance of *A. thaliana* transformants expressing the *Sspdhx* RNAi construct, 5‐week‐old *A. thaliana* T_2_ transformants were inoculated with mycelial plugs (2 mm in diameter). Following incubation at 20°C for 36 h, lesion areas were measured.

### 4.12 Prediction of Potential Off‐Targets in Different Species

To assess whether the dsRNA fragment targeting *Sspdhx* could unintentionally silence genes in host plants or non‐pathogenic fungi, the sequence used in both SIGS and HIGS experiments was analysed for potential off‐targets using siFi21 (Luck et al. 2019). si‐Fi (siRNA‐Finder) predicts RNAi efficiency and specificity by evaluating candidate siRNAs' thermodynamic asymmetry, local mRNA accessibility, and database sequence matches, reporting the number of perfect matches (mis0) for each transcript.

Analyses were performed against RefSeq mRNA databases for host plants and selected pathogens/non‐pathogens with available transcripts, and against genome sequences for species lacking curated transcriptomes, including *Stromatinia cepivora*, *Sclerotinia trifoliorum*, and *Monilinia fructicola* .

### 4.13 Effect of *Sspdhx*‐dsRNA on the Growth of Biocontrol Fungi

To evaluate the potential off‐target effects of *Sspdhx*‐dsRNA, three common biocontrol fungi, *B. bassiana*, *C. minitans*, and *T. harzianum*, were inoculated on PDA supplemented with *Sspdhx*‐dsRNA (50 ng/μL). The fungi were cultured under their respective optimal temperatures: *B. bassiana* at 25°C for 7 days, *C. minitans* at 20°C for 7 days, and *T. harzianum* at 20°C for 2 days. After incubation, colony diameters were measured, and mycelia were harvested for total RNA extraction. RT‐qPCR was subsequently performed to examine whether *Sspdhx*‐dsRNA influenced the expression of *Sspdhx* homologues in these non‐target biocontrol fungi.

## Author Contributions

**Qingna Shang:** methodology, software, data curation, investigation, formal analysis, visualisation, writing – original draft. **Shunrui Yang:** data curation, investigation, validation, formal analysis. **Chunyu Feng:** methodology, data curation, investigation, validation, formal analysis. **Chong Xie:** methodology, data curation, investigation, visualisation. **Yunshu Song:** investigation, validation, formal analysis. **Jiatao Xie:** conceptualization, supervision, funding acquisition, resources, writing – review and editing. **Yanping Fu:** supervision, visualisation, resources. **Jiasen Cheng:** supervision, visualisation, resources. **Qing Cai:** supervision, visualisation, resources. **Bo Li:** supervision, funding acquisition, resources. **Tao Chen:** supervision, funding acquisition, resources. **Xiao Yu:** supervision, funding acquisition, resources. **Yang Lin:** supervision, funding acquisition, resources. **Daohong Jiang:** supervision, funding acquisition, resources, writing – review and editing. **Xueqiong Xiao:** conceptualization, formal analysis, supervision, funding acquisition, project administration, resources, writing – review and editing.

## Funding

This work was supported by National Natural Science Foundation of China, 32372495, 32072372; the earmarked fund for the China Agriculture Research System CARS‐12.

## Conflicts of Interest

The authors declare no conflicts of interest.

## Supporting information


**Figure S1:** Generation and verification of the Δ*Sspdhx* mutants. (a) Schematic diagram of the *Sspdhx* gene knockout strategy. (b) PCR verification of the Δ*Sspdhx* mutants and complemented strain. Amplicons correspond to the upstream (U) and downstream (D) flanking regions, the hygromycin resistance gene (H), and an internal fragment of the *Sspdhx* gene (G). (c) Southern blot validation of the Δ*Sspdhx* mutants. The *λ‐*HindIII digest was used as marker. Linearised plasmid pUCH18, which carries the hygromycin resistance gene, served as a positive control. Digested Sunf‐M genomic DNA was used as a negative control. (d) Reverse transcription‐PCR was performed to analyse the transcriptional level of *Sspdhx* in different strains, confirming the successful deletion of *Sspdhx*. The *β‐tubulin* gene of *S. sclerotiorum* was used as an internal reference. The primers used are listed in Table S1.

**Figure S2:** The *Sspdhx* deletion mutants exhibit increased sensitivity to H_2_O_2_. (a) Effects of different H_2_O_2_ concentrations on colony morphology and growth rate of wild‐type strain Sunf‐M, Δ*Sspdhx* mutants, and the complemented strain *Sspdhx‐19C*. Scale bar, 1 cm. (b) Inhibition rates of growth of strain Sunf‐M, *Sspdhx* deletion mutants and *Sspdhx‐19C* under different H_2_O_2_ concentrations. All data were analysed using one‐way ANOVA and error bars indicate the standard error. Asterisks (*) denote significant differences, (*p < 0.05, **p < 0.01, ***p < 0.001, ****p < 0.0001), ns indicates no significant difference.

**Figure S3:** Growth of the wild‐type strain Sunf‐M, Δ*Sspdhx* mutants, and the complemented strain *Sspdhx‐19C* of *Sclerotinia sclerotiorum* under stress conditions. (a) Colony morphology of Sunf‐M, Δ*Sspdhx* mutants, and *Sspdhx‐19C* grown on potato dextrose agar (PDA) or PDA supplemented with Congo red (3 mg/mL), SDS (0.01%), CaCl_2_ (0.5 M), or NaCl (1 M). (b) Inhibition rates of mycelial growth of Sunf‐M, Δ*Sspdhx* mutants, and *Sspdhx‐19C* under the indicated stress conditions. All data were analysed using one‐way ANOVA and error bars indicate the standard error. Asterisks (*) denote significant differences, (**p < 0.01, ***p < 0.001, ****p < 0.0001), ns indicates no significant difference.

**Figure S4:** Overview of RNA‐seq data analysis of Sunf‐M and Δ*Sspdhx* mutants during vegetative growth and host infection. (a) Principal component analysis (PCA) of transcriptomic profiles. (b) Correlation analysis of gene expression among biological replicates. (c) Volcano plot of differentially expressed genes (DEGs) in Δ*Sspdhx* relative to Sunf‐M during vegetative growth. (d) Volcano plot of DEGs in Δ*Sspdhx* relative to Sunf‐M during host infection. (e) Gene Ontology (GO) enrichment analysis of DEGs during vegetative growth. (f) Gene Ontology (GO) enrichment analysis of DEGs during host infection.

**Figure S5:** Reverse transcription‐quantitative PCR analysis of selected genes in Sunf‐M and Δ*Sspdhx* mutant. The *Sclerotinia sclerotiorum β‐tubulin* gene was used as an internal reference. Statistical analysis was performed using a Student's *t* test. Asterisks indicate significant differences compared with Sunf‐M (*p < 0.05, **p < 0.01).

**Table S1:** Primers used in this study.

**Table S2:** Off‐target assessment of the *Sspdhx* fragment for spray‐induced gene silencing (SIGS) and host‐induced gene silencing (HIGS).

## Data Availability

All transcriptomics data have been deposited in the Sequence Read Archive (SRA) on NCBI (https://www.ncbi.nlm.nih.gov/) and are openly available with the dataset identifiers PRJNA1417617.
