# Supplementary material for: Sspdhx Related to the Development and Virulence of Sclerotinia sclerotiorum Represents a Potential RNAi Target for Controlling Sclerotinia Disease
Source: Mol Plant Pathol. 2026 Mar 16;27(3):e70244. doi: 10.1111/mpp.70244 (PMC13097459; doi:10.1111/mpp.70244)
Supplement: Supplementary file 1 — Figure S1: Generation and verification of the ΔSspdhx mutants. (a) Schematic diagram of the Sspdhx gene knockout strategy. (b) PCR verification of the ΔSspdhx mutants and complemented strain. Amplicons correspond to the upstream (U) and downstream (D) flanking regions, the hygromycin resistance gene (H), and an internal fragment of the Sspdhx gene (G). (c) Southern blot validation of the ΔSspdhx mutants. The λ‐HindIII digest was used as marker. Linearised plasmid pUCH18, which carries the hygromycin resistance gene, served as a positive control. Digested Sunf‐M genomic DNA was used as a negative control. (d) Reverse transcription‐PCR was performed to analyse the transcriptional level of Sspdhx in different strains, confirming the successful deletion of Sspdhx. The β‐tubulin gene of S. sclerotiorum was used as an internal reference. The primers used are listed in Table S1. [file MPP-27-e70244-s001.docx]

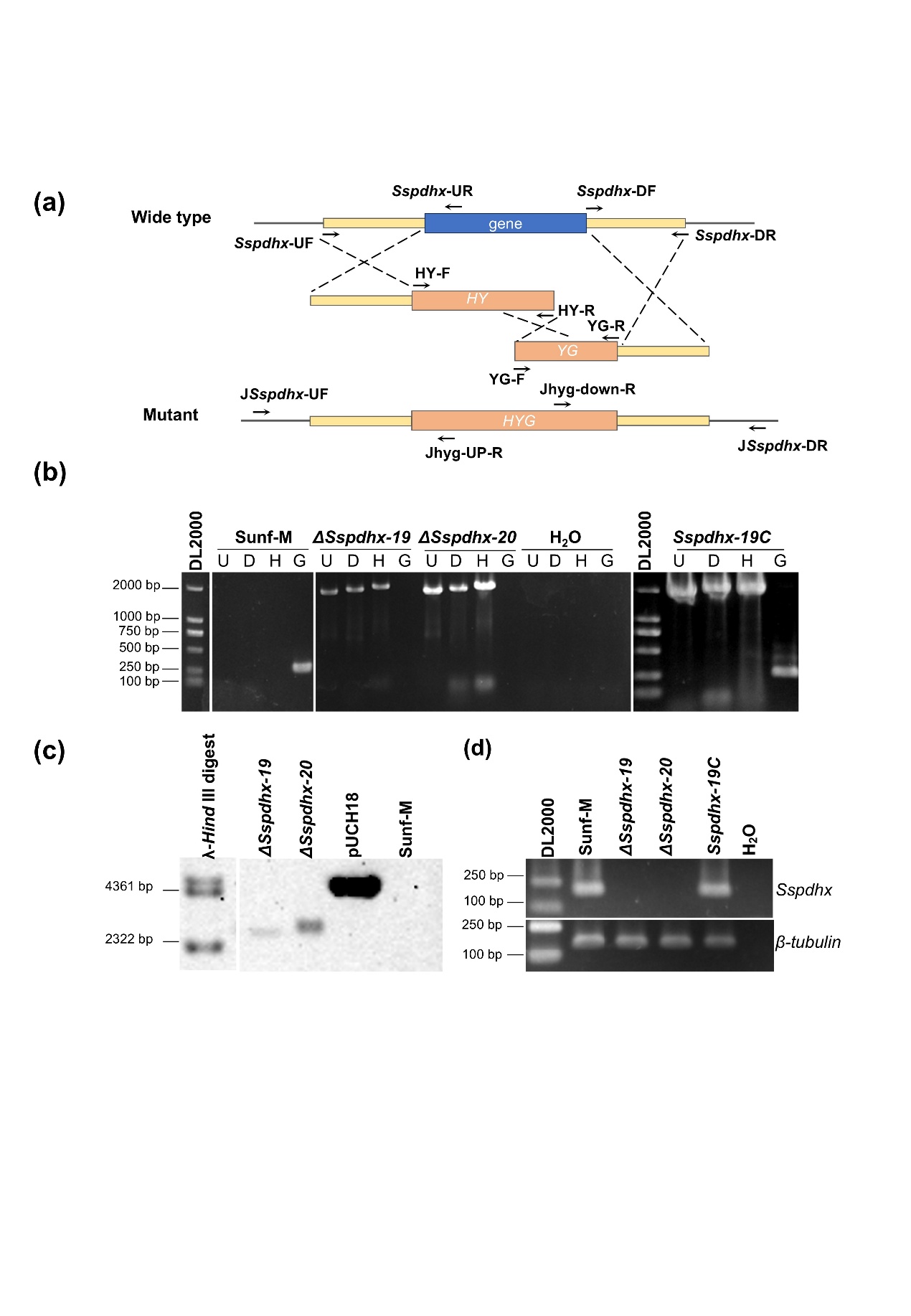


Figure S1. Generation and verification of the *ΔSspdhx* mutants. (a) Schematic diagram of the *Sspdhx* gene knockout strategy. (b) PCR verification of the *ΔSspdhx* mutants and complemented strain. Amplicons correspond to the upstream (U) and downstream (D) flanking regions, the hygromycin resistance gene (H), and an internal fragment of the *Sspdhx* gene. (G). (c) Southern blot validation of the *ΔSspdhx* mutants. The *λ*-*Hind* III digest was used as marker. Linearized plasmid pUCH18, which carries the hygromycin resistance gene, served as a positive control. Digested Sunf-M genomic DNA was used as a negative control. (d) RT-PCR was performed to analyze the transcriptional level of *Sspdhx* in different strains, confirming the successful deletion of Sspdhx. The *β-tubulin* gene of *S. sclerotiorum* was used as an internal reference. The primers used are listed in Table S1.
