# Supplementary material for: Sspdhx Related to the Development and Virulence of Sclerotinia sclerotiorum Represents a Potential RNAi Target for Controlling Sclerotinia Disease
Source: Mol Plant Pathol. 2026 Mar 16;27(3):e70244. doi: 10.1111/mpp.70244 (PMC13097459; doi:10.1111/mpp.70244)
Supplement: Supplementary file 2 — Figure S2: The Sspdhx deletion mutants exhibit increased sensitivity to H2O2. (a) Effects of different H2O2 concentrations on colony morphology and growth rate of wild‐type strain Sunf‐M, ΔSspdhx mutants, and the complemented strain Sspdhx‐19C. Scale bar, 1 cm. (b) Inhibition rates of growth of strain Sunf‐M, Sspdhx deletion mutants and Sspdhx‐19C under different H2O2 concentrations. All data were analysed using one‐way ANOVA and error bars indicate the standard error. Asterisks (*) denote significant differences, (*p < 0.05, **p < 0.01, ***p < 0.001, ****p < 0.0001), ns indicates no significant difference. [file MPP-27-e70244-s007.docx]

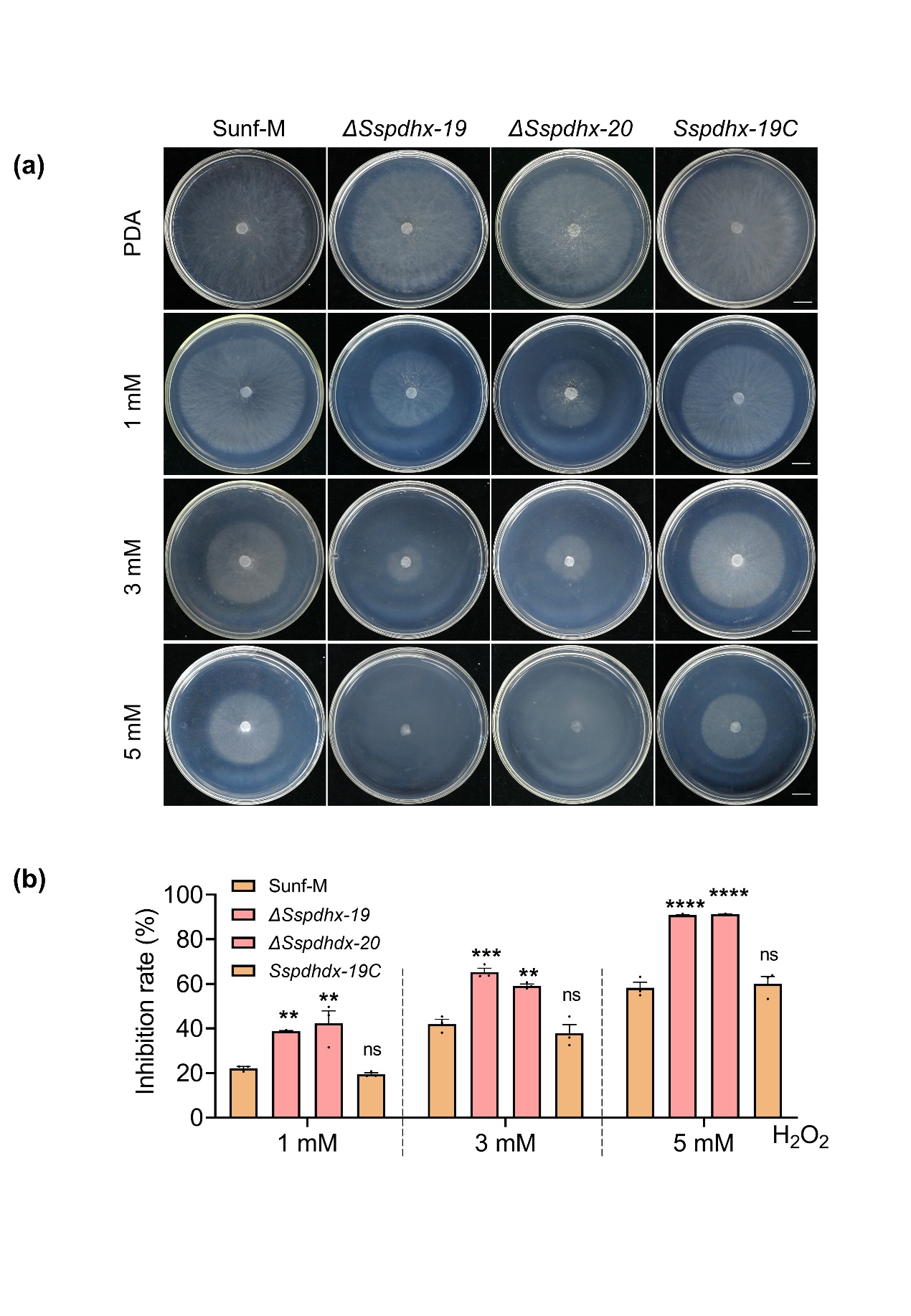


Figure S2. The *Sspdhx* deletion mutants exhibit increased sensitivity to H₂O₂. (a) Effects of different H₂O₂ concentrations on colony morphology and growth rate of wild-type strain Sunf-M, *ΔSspdhx* mutants, and the complemented strain *Sspdhx-19C*. Scale bar, 1 cm. (b) Inhibition rates of growth of strain Sunf-M, *Sspdhx* deletion mutants and *Sspdhx-19C* under different H₂O₂ concentrations. All data were analyzed using one-way ANOVA and error bars indicate the standard error. Asterisks (*) denote significant differences, (**P* < 0.05, ***P* < 0.01, ****P* < 0.001, *****P* < 0.0001), ns indicates no significant difference.
