# Supplementary material for: Sspdhx Related to the Development and Virulence of Sclerotinia sclerotiorum Represents a Potential RNAi Target for Controlling Sclerotinia Disease
Source: Mol Plant Pathol. 2026 Mar 16;27(3):e70244. doi: 10.1111/mpp.70244 (PMC13097459; doi:10.1111/mpp.70244)
Supplement: Supplementary file 3 — Figure S3: Growth of the wild‐type strain Sunf‐M, ΔSspdhx mutants, and the complemented strain Sspdhx‐19C of Sclerotinia sclerotiorum under stress conditions. (a) Colony morphology of Sunf‐M, ΔSspdhx mutants, and Sspdhx‐19C grown on potato dextrose agar (PDA) or PDA supplemented with Congo red (3 mg/mL), SDS (0.01%), CaCl2 (0.5 M), or NaCl (1 M). (b) Inhibition rates of mycelial growth of Sunf‐M, ΔSspdhx mutants, and Sspdhx‐19C under the indicated stress conditions. All data were analysed using one‐way ANOVA and error bars indicate the standard error. Asterisks (*) denote significant differences, (**p < 0.01, ***p < 0.001, ****p < 0.0001), ns indicates no significant difference. [file MPP-27-e70244-s006.docx]

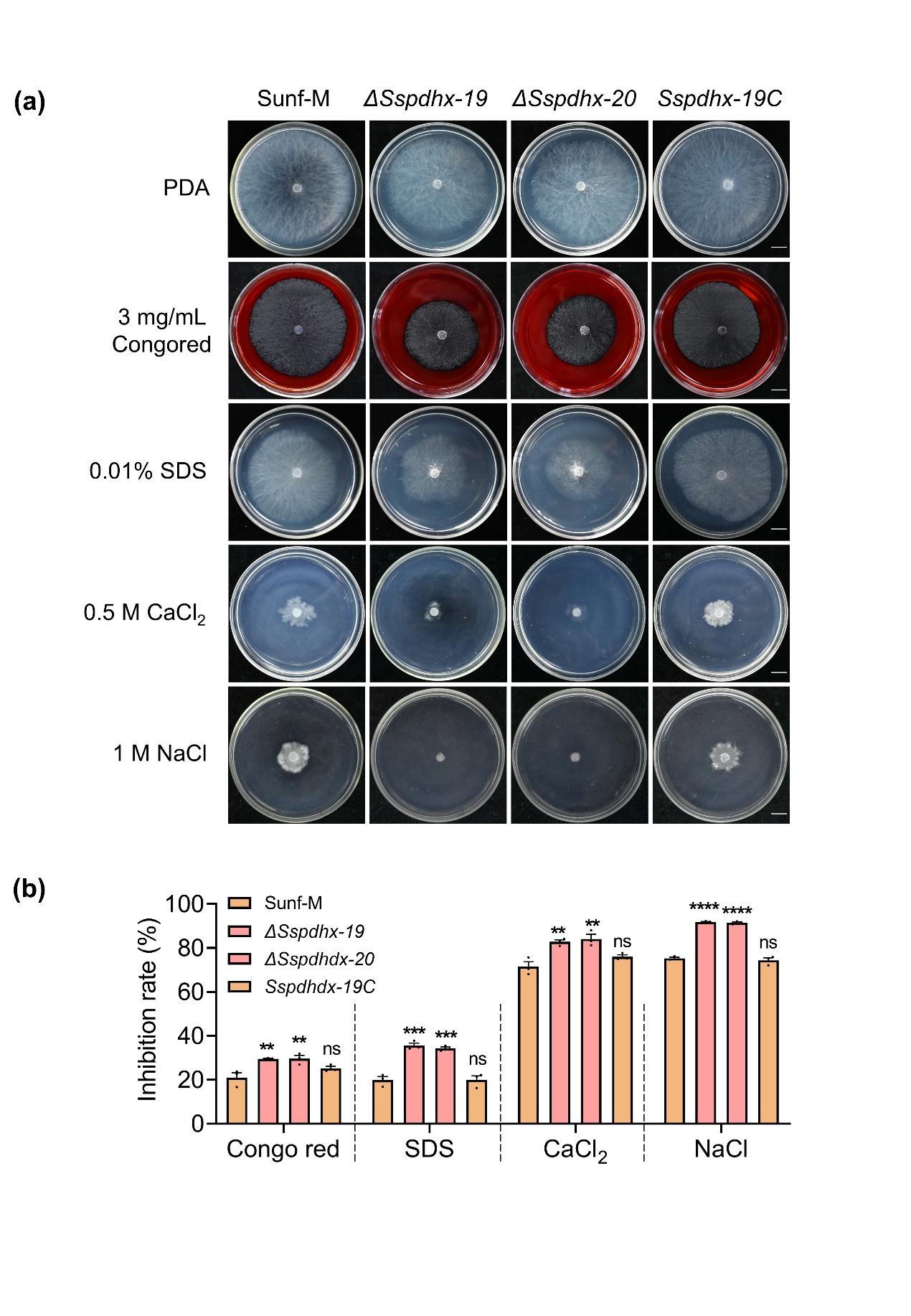


Figure S3. Growth of the wild-type strain Sunf-M, *ΔSspdhx* mutants, and the complemented strain *Sspdhx-19C* of *S. sclerotiorum* under stress conditions. (a) Colony morphology of Sunf-M, *ΔSspdhx* mutants, and *Sspdhx-19C* grown on PDA or PDA supplemented with Congo red (3 mg / mL), SDS (0.01%), CaCl₂ (0.5 M), or NaCl (1 M). (b) Inhibition rates of mycelial growth of Sunf-M, *ΔSspdhx* mutants, and *Sspdhx-19C* under the indicated stress conditions. All data were analyzed using one-way ANOVA and error bars indicate the standard error. Asterisks (*) denote significant differences, (***P* < 0.01, ****P* < 0.001, *****P* < 0.0001), ns indicates no significant difference.
