# Supplementary material for: Sspdhx Related to the Development and Virulence of Sclerotinia sclerotiorum Represents a Potential RNAi Target for Controlling Sclerotinia Disease
Source: Mol Plant Pathol. 2026 Mar 16;27(3):e70244. doi: 10.1111/mpp.70244 (PMC13097459; doi:10.1111/mpp.70244)
Supplement: Supplementary file 4 — Figure S4: Overview of RNA‐seq data analysis of Sunf‐M and ΔSspdhx mutants during vegetative growth and host infection. (a) Principal component analysis (PCA) of transcriptomic profiles. (b) Correlation analysis of gene expression among biological replicates. (c) Volcano plot of differentially expressed genes (DEGs) in ΔSspdhx relative to Sunf‐M during vegetative growth. (d) Volcano plot of DEGs in ΔSspdhx relative to Sunf‐M during host infection. (e) Gene Ontology (GO) enrichment analysis of DEGs during vegetative growth. (f) Gene Ontology (GO) enrichment analysis of DEGs during host infection. [file MPP-27-e70244-s004.docx]

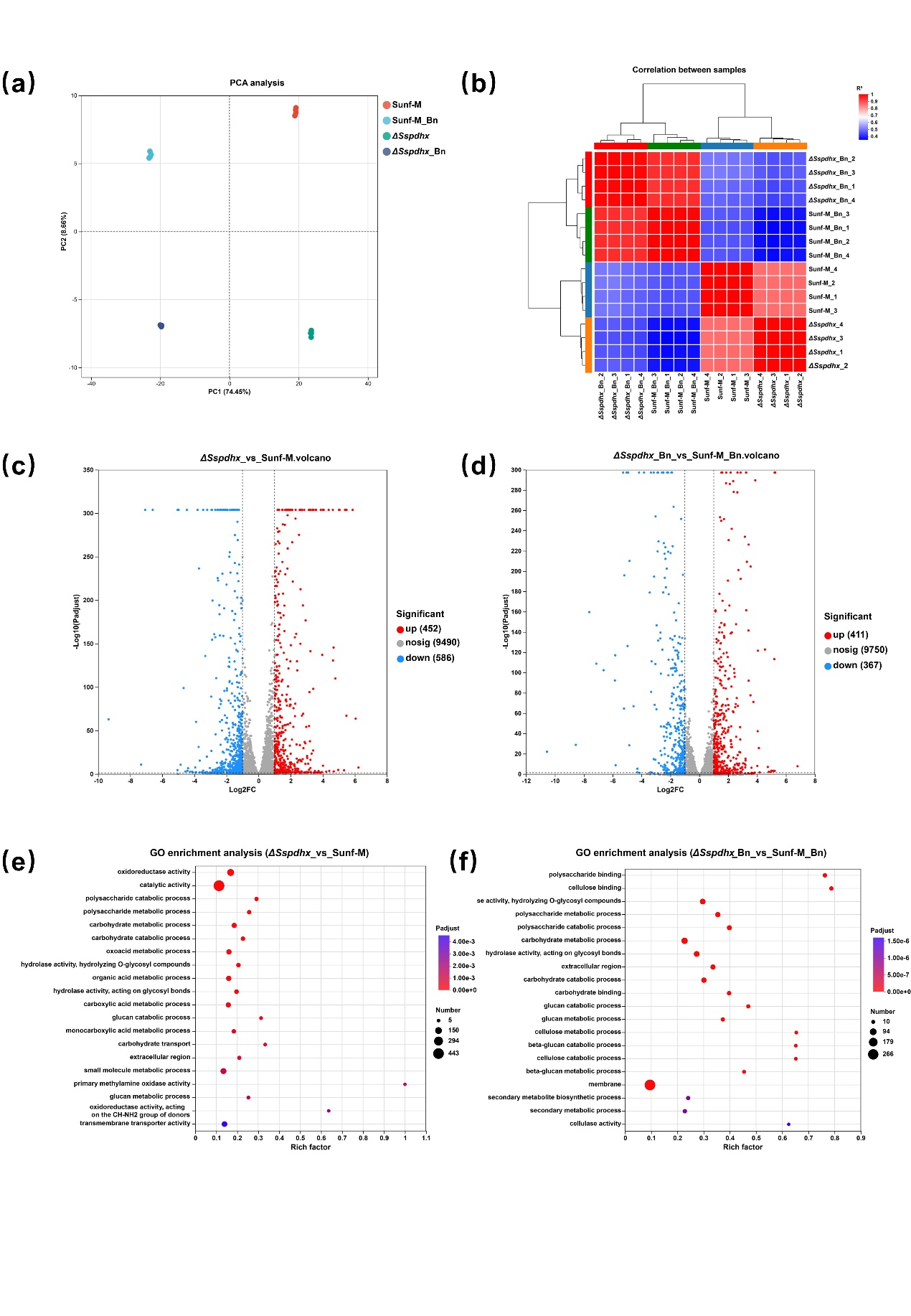


Figure S4. Overview of RNA-seq data analysis of Sunf-M and *ΔSspdhx* mutants during vegetative growth and host infection. (a) Principal component analysis (PCA) of transcriptomic profiles. (b) Correlation analysis of gene expression among biological replicates. (c) Volcano plot of differentially expressed genes (DEGs) in *ΔSspdhx* relative to Sunf-M during vegetative growth. (d) Volcano plot of DEGs in *ΔSspdhx* relative to Sunf-M during host infection. (e) Gene Ontology (GO) enrichment analysis of DEGs during vegetative growth. (f) Gene Ontology (GO) enrichment analysis of DEGs during host infection.
