# Supplementary material for: Sspdhx Related to the Development and Virulence of Sclerotinia sclerotiorum Represents a Potential RNAi Target for Controlling Sclerotinia Disease
Source: Mol Plant Pathol. 2026 Mar 16;27(3):e70244. doi: 10.1111/mpp.70244 (PMC13097459; doi:10.1111/mpp.70244)
Supplement: Supplementary file 5 — Figure S5: Reverse transcription‐quantitative PCR analysis of selected genes in Sunf‐M and ΔSspdhx mutant. The Sclerotinia sclerotiorum β‐tubulin gene was used as an internal reference. Statistical analysis was performed using a Student's t test. Asterisks indicate significant differences compared with Sunf‐M (*p < 0.05, **p < 0.01). [file MPP-27-e70244-s002.docx]

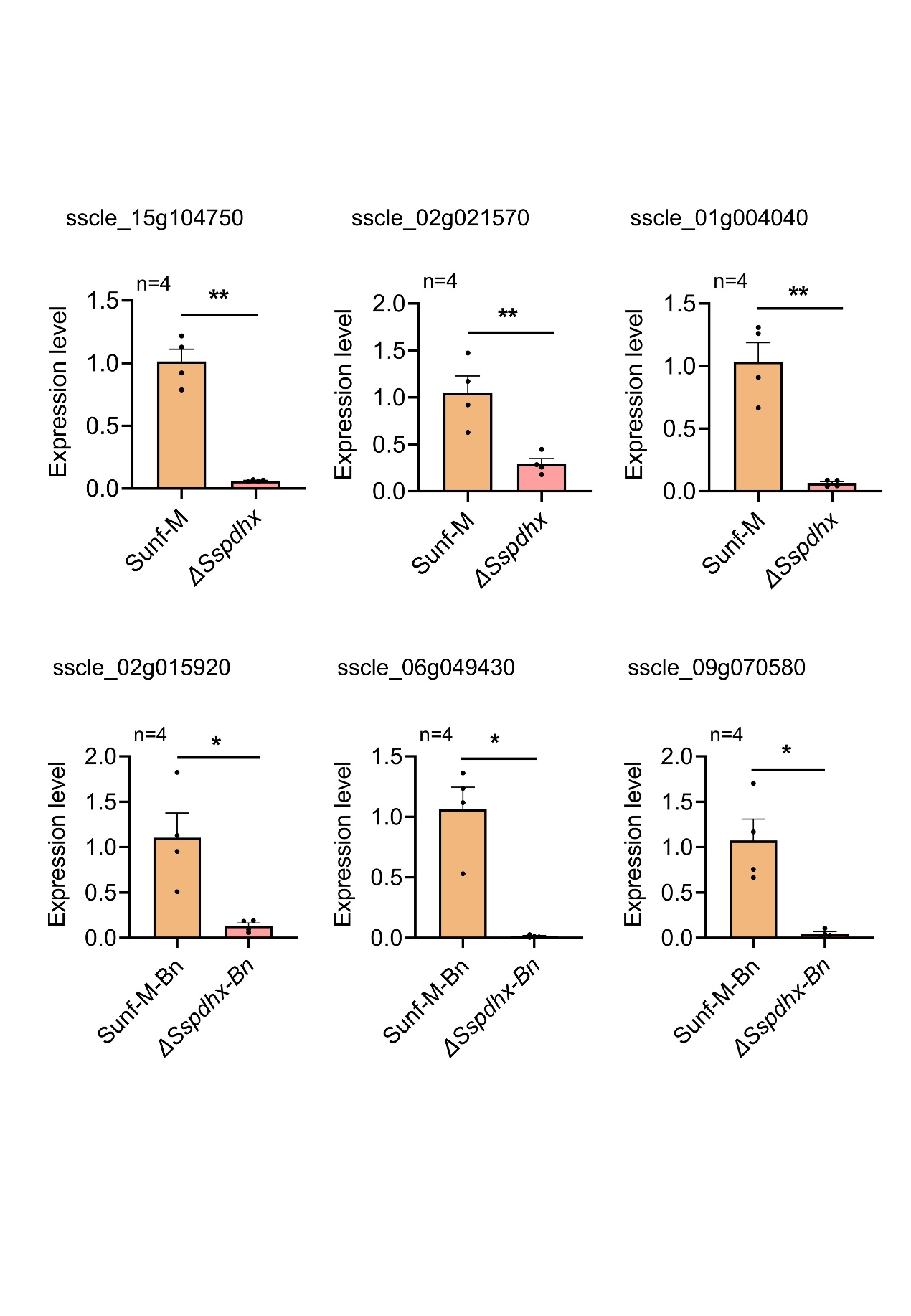


Figure S5. RT-qPCR analysis of selected genes in Sunf-M and *ΔSspdhx* mutant. The *S. sclerotiorum* β-tubulin gene was used as an internal reference. Statistical analysis was performed using a Student’s t-test. Asterisks indicate significant differences compared with Sunf-M (**P* < 0.05, ***P* < 0.01).
